# Beneficial Effects of Physical Activity in Diabetic Patients

**DOI:** 10.3390/jfmk5030070

**Published:** 2020-09-04

**Authors:** Francesca Cannata, Gianluca Vadalà, Fabrizio Russo, Rocco Papalia, Nicola Napoli, Paolo Pozzilli

**Affiliations:** 1 Department of Endocrinology and Diabetes, Campus Bio-Medico University of Rome, Via Alvaro del Portillo 21, 00128 Rome, Italy; n.napoli@unicampus.it (N.N.); p.pozzilli@unicampus.it (P.P.); 2 Department of Orthopaedic and Trauma Surgery, Campus Bio-Medico University of Rome, Via Alvaro del Portillo 21, 00128 Rome, Italy; g.vadala@unicampus.it (G.V.); fabrizio.russo@unicampus.it (F.R.); r.papalia@unicampus.it (R.P.)

**Keywords:** diabetes, physical activity, exercise, non-pharmacological therapy

## Abstract

One of the main goals of diabetic therapy is to achieve the best metabolic control to prevent the development and progression of potential complications. A multidisciplinary approach characterized by the combination of diet, physical activity (PA) and drug therapy with oral and injectable (non-insulin) pharmacological agents, is desirable to optimize metabolic control. The aim of this review is to explain the contribution of PA and its beneficial effects on patients affected by type 1 (T1D) and type 2 diabetes (T2D). We provide an overview of evidence on the effects of PA for the main two types of diabetes mellitus (DM) to identify the right level of PA to be recommended. We discuss the physiological and clinical role of PA in people with DM. It can be concluded that the objective of antidiabetic therapy should be the achievement and optimization of metabolic control through a multidisciplinary approach involving non-pharmacological therapy such as diet and PA, which has a crucial role.

## 1. Introduction

According to the World Health Organization (WHO) diabetes mellitus (DM) is defined as a group of metabolic disorders with different aetiologies characterized by chronic hyperglycaemia associated with alterations in glucose, lipid and protein metabolisms secondary to defects in insulin secretion, action or both. The prevalence of DM has reached epidemic proportions. Currently, almost 390 million individuals worldwide are affected, and more than 590 million individuals are expected to develop this condition by 2035. At the same time, more than half of the diabetic population remains undiagnosed and therefore untreated [1].

Chronic hyperglycaemia is associated with damage, dysfunction and collapse of different anatomical districts [2]. Therefore, continuous interventions to correct blood glucose levels and cardiovascular risk factors are crucial for preventing acute and chronic complications [3].

The non-pharmacological therapy of DM is mainly focused on lifestyle changes in terms of physical activity (PA), diet and smoking habit [4]. Adequate lifestyle changes have beneficial effects on the reduction of anthropometric parameters such as body weight, body mass index (BMI), waist circumference, and also blood parameters related to fat and glucose profiles [5]. Moreover, in people with diabetes, regular PA potentially reduces the amount and dose of antidiabetic therapy and insulin dosage [6].

PA is defined as a subgroup of activities referred to all repetitive, planned and structured movements specifically designed to improve health and physical fitness [7]. Aerobic exercise consists of rhythmic, repeated and continuous movements of the same large muscle groups for at least 10 min [8]. Exercise against resistance consists of activities that use muscle strength to work against a load that offers resistance [9]. The physical fitness term, refers to a series of attributes that can be achieved by training, such as endurance and strength, abilities that are closely related to PA [5]. However, PA in overweight or obese individuals with diabetes often represents an insurmountable problem since these subjects suffer of several musculoskeletal disorders such as osteoarthritis [10], chronic low back pain due to intervertebral disc degeneration [11], and other musculoskeletal disorders [12,13].

The aim of this review is to investigate the beneficial effects of PA in patients affected by type 1 diabetes (T1D) and type 2 diabetes (T2D) through the evaluation and analysis of literature to improve exercise prescription in these delicate population.

## 2. Type of Physical Activity in Type 1 Diabetes and Type 2 Diabetes Subjects

Several studies have investigated the effects of different types of PA in people with diabetes. Nevertheless, the heterogeneity of these studies suggests that there is no clear evidence. The differences mainly concern the type of PA; its characteristics; the outcomes studied; acute or chronic effects and the pharmacological approach before, during and after PA.

Major types of PA studied in these subjects include aerobic, anaerobic and high-intensity interval training (HIIT).

Aerobic PA, or endurance PA, is characterized by repeated and continuous movement of large muscle groups. Examples of aerobic exercise include cycling, dancing, hiking, jogging/long distance running, swimming and walking [14]. Aerobic exercise activates glycolysis leading to a rapid production of ATP and lactate. In individuals with diabetes, it has been proved to be able to improve insulin sensitivity, lung, immune and cardiovascular function [14], and it is associated with lower risk of cardiovascular diseases and overall mortality [15]. In particular, aerobic exercise improves lipid metabolism and decreases insulin resistance in T1D and reduces blood pressure, triglycerides, insulin resistance and glycated haemoglobin (HbA1C) in T2D [16]. Recommendations suggest that aerobic activity should last at least 150 min/week at moderate to vigorous intensity in people with diabetes [17]. However, aerobic exercise can be performed continuously or as HIIT, which is characterized by short intense bursts with recovery periods interspersed. Similar cardioprotective and metabolic benefits can be obtained by HIIT in younger or more physically fit patients when vigorously performed for 75 min/week [18]. However, HIIT is mainly recommended in clinically stable patients who already perform intense physical activities and under supervision [19].

Resistance or strength training includes exercises with free or body weights, machines or elastic bands. Resistance exercises are able to increase muscle mass and strength, through the induction of muscle hypertrophy and neuromuscular remodelling, and also improvements in physical function, mental and cardiovascular health, insulin sensitivity and lipid metabolism [14]. The effect of resistance exercise on T1D patients is related to the improvement in muscular strength and lipid profile, a better control of blood glucose levels and reduced dose of insulin [20]. In T2D patients, resistance training improves blood pressure and increases muscle mass and strength, which may positively impact insulin responsiveness and metabolic control [21]. Indeed, several randomized clinical studies have shown that metabolic control, lipid and cardiovascular disease risk profile can be enhanced in patients with T2D through resistance training [22]. Recommendations suggest engaging in 2–3 non-consecutive days/week of resistance exercise for adults affected by DM using a variety of strength training modalities [17].

Flexibility exercises, can improve range of motion around joints through stretching, and balance activities can enhance balance and gait preventing falls in older adults [23]. Reduced joint mobility, which may be due to advanced glycation end products, is often found in older individuals with diabetes [24]. Therefore, it is recommended to perform both flexibility and balance activities for 2 or more sessions/week, especially by older adults with peripheral neuropathy [14]. However, flexibility activities do not affect glucose control or insulin action, and they should not replace other recommended exercises such as aerobic and resistance training [25]. A combination of balance, flexibility and resistance activities is represented by Tai Chi and Yoga, which can be performed based on individual preferences. Yoga can help the metabolic control, lipid profile and body composition in T2D patients [26]. On the other hand, Tai Chi in T2D patients with neuropathy can improve neurologic symptoms, balance but also glucose control and quality of life [27].

However, it is crucial to personalize the exercise program according to individual’s health status, physical function, exercise responses and goals. Patients who are unable or unwilling to perform PA as recommended can still benefit from exercising at lower levels or reducing total time engaged in sedentary activities. In Table 1 are summarized differences in PA types and their recommendation in T1D and T2D subjects.

## 3. Beneficial Effects of Physical Activity in Type 1 Diabetes

T1D approximately accounts for 5–10% of all people with diabetes. It is related to genetic and environmental factors, even though the latter are still poorly defined [28], and it is characterized by an autoimmune and cell-mediated destruction of pancreatic β-cells leading to insulin deficiency with a tendency to ketoacidosis. The β-cell destruction rate is relatively variable, rapid in childhood and slower in adults [29].

Regular PA in people with T1D produces the same positive effects as for non-diabetics in term of morbidity and mortality. Clinical and experimental studies have shown that the benefits of PA in subjects with T1D are mainly related to the (1) increased insulin sensitivity in skeletal muscle, (2) possible positive effects on glycaemic control, (3) increased antioxidant defences and reduced oxidation, (4) decreased blood pressure, (5) reduction of cardiovascular diseases, (6) optimization of lipid profile and (7) enhancement of renal function [30].

However, one of the main obstacles in regular PA is the fear of hypoglycaemia. Prevention of hypoglycaemia during and after PA remains an important issue in the management of T1D therapy.

Aerobic PA involves several physiological adaptations. Indeed, it can lead to a greater capillary density, greater expression and translocation of GLUT4 towards the plasma membrane, an increased number of muscle fibres which are more sensitive to the action of insulin, changes in the composition of phospholipidic proteins on sarcolemma, an increased activity of glycolytic and oxidative enzymes and an increased use of muscle glycogen [31]. Ebeling et al. evaluated the insulin sensitivity in skeletal muscles of 11 athletes with T1D who participated in athletic competitions compared to 12 sedentary individuals with diabetes. Glycaemic control, insulin uptake throughout the body and forearm, oxidation of glucose, lipids and muscle glycogen and GLUT4 concentrations were measured. Glucose levels and its oxidation were similar in both groups, while both energy expenditure and lipid oxidation increased in athletes. Lipidic oxidation was inversely related to glycogenosynthesis activity; muscle glycogen and GLUT4 activity were not different in the two groups [32]. Jimenez et al. examined the acute effect of resistance exercise on insulin sensitivity in subjects with T1D [33]. The insulin sensitivity was not significantly different between the trained and untrained group. They concluded that a single period of resistance training does not alter the sensitivity of insulin in people with T1D. Adequate PA can reduce morbidity and mortality in this population [34]. However, to achieve these benefits, individuals with T1D require adjustments in insulin doses.

Patients affected by T1D with poor glycaemic control have higher levels of triglycerides than non-diabetic subjects. Glycaemic control is the main factor interfering with lipid concentration in patients with DM. The benefits of PA on the lipid profile in subjects affected by T1D have been demonstrated, suggesting that this non-pharmacological approach represents an additional alternative therapy [35]. Lehmann et al. showed a better lipid profile, independently from the glycaemic control, in adolescents with T1D who have joined a dietary and training program [36]. Further studies have shown an improvement in lipid profile after physical training in subjects with T1D characterized by a reduction in total cholesterol, LDL cholesterol and triglyceride levels and an increase in HDL cholesterol [37]. Austin et al. reported a reduction or maintenance of levels of high-density lipoprotein LP(a), a cardiovascular risk factor, after a period of physical conditioning in T1D patients [35].

Fascinating research has assessed the impact of PA on cardiovascular risk factors in subjects with T1D, focusing on glycaemic control and plasma lipids (HDL cholesterol, triglycerides, Lp(a) lipoprotein), blood pressure, weight control and abdominal fat. The research emphasized various measures to be implemented during endurance training in patients with T1D. A total of 20 subjects with T1D were examined, engaged in a training program for a period of three months. The most practiced sports were cycling, long-distance running and trekking. The type and time of this activity was recorded, and the subjects performed training sessions lasting at least 135 min per week. The study showed that increasing PA is safe and does not necessarily result in hypoglycaemic episodes. It was shown a linear correlation between abdominal fat loss and decreased blood pressure, both cardiovascular risk factors [36].

The effects of PA on blood pressure and the cardiorespiratory system in subjects with T1D have also been widely evaluated [38]. For the first time, a study demonstrated that patients with T1D who followed an aerobic exercise program for 3 months decreased their blood pressure and heart rate as well as improved their lipid profile [39]. Mosher et al. carried out a study to evaluate the effects of a “circuit” aerobic training on the cardiorespiratory system, muscular system, regulation of glucose and the concentration of lipids. A total of 10 adolescents with T1D and 10 non-diabetic adolescents performed a physical training including mixed endurance and strength exercises for 3 times a week for 12 weeks [40]. Circuit aerobic training in teenagers with T1D improved their cardiorespiratory endurance, muscle strength, lipid profile and glucose regulation [41].

PA also significantly increases urinary excretion by increased albuminuria [42]. This physiological response is to be considered as an indicator of early diabetic nephropathy. An important role is played by the workload, which during PA could modify the albumin values [43]. Individuals with and without diabetes were compared to those who had urinary albumin excretion after physical stress, and then, the differences between these two groups of individuals were identified [44]. The long-term effect of PA on urinary excretion of albumin in patients with and without diabetes is still unclear. Recent studies have shown that 10 weeks of physical training on the treadmill reduced polyuria and glycosuria in patients with DM without significantly reducing proteinuria [45]. These data suggest that aerobic physical training improves the metabolic profile but does not promote important benefits in diabetic nephropathy when evaluated by proteinuria.

PA has been also recognized as a way to increase antioxidant defences and reduce oxidative stress and blood pressure levels, suggesting beneficial mechanisms that could act in reducing kidney injury [46]. Harmer et al. evaluated the effects of sprint training on potassium regulation and muscle ATPase in subjects with T1D, compared to people without diabetes. This study shows that subjects with T1D can safely perform intense PA while maintaining potassium concentrations; however, its concentration will depend on adequate insulin administration during recovery. However, the lack of correlation between the potassium content in plasma and sodium content may indicate that different mechanisms controlling potassium concentration are compromised [47]. Several clinical studies have evaluated the antioxidant effect of PA in T1D subjects. Woo et al. evaluated the activity of antioxidant enzymes and their effect on DNA. The study compared the effect of a 12-week training program on a group of children with T1D and a healthy control group. Although, the training program increased the antioxidant enzyme activity, the training with low-intensity aerobic exercises over a 12-week period accelerated the defensive effects of the antioxidant enzymes in children with T1D [48].

**Figure 1:**
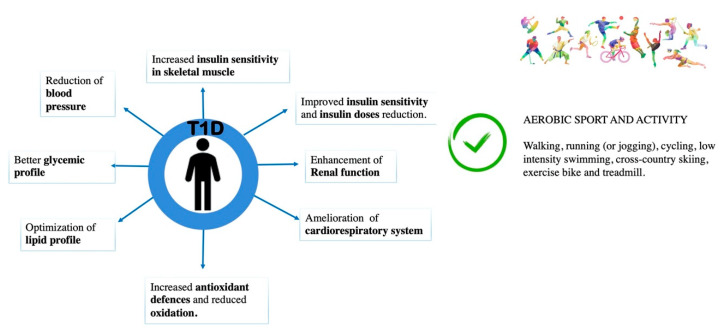
Beneficial effects of PA and recommended sports for T1D subjects.

Major beneficial effects of PA and recommended sports in T1D subjects are shown in Figure 1.

## 4. Beneficial Effect of Physical Activity in Type 2 Diabetes

T2D affects 90–95% of individuals with diabetes, and it is characterized by insulin resistance and often by a relative insulin deficiency; however, specific aetiology is still unclear. Several T2D subjects are affected by obesity, which is a leading cause of insulin resistance.

PA is a key component of the therapeutically approach for T2D subjects. Indeed, it has positive effects on (1) reduction of body weight and body mass index (BMI), (2) improvement in glucose tolerance and insulin sensitivity, (3) reduction of HbA1c level, (4) improvement of cardiorespiratory system, (5) reduction of cardiovascular disease risk (CVD) and (6) reduction of incidence of new cases of diabetes [17,49].

Current recommendations for T2D subjects are to perform 150 min of moderate intensity PA every day of the week [50]. However, given the length and effects of PA on insulin sensitivity of about 24 to 72 h, it is advisable to practice PA with recovery intervals of no more than 3 days [51]. Most clinical studies that have evaluated PA in these patients have used a frequency of three times a week, and many people find it easier to plan fewer sessions [52].

Unfortunately, despite the potential benefits, many people with diabetes are completely sedentary or unable to increase their PA level. Indeed, overweight and obesity cause an increase in the abdominal fat distribution that can contribute to the development of insulin resistance [53]. Rogers et al. investigated the effects of PA on insulin resistance reporting that a 7-day exercise program of moderate intensity can improve glucose tolerance without changes in body weight. However, it is also able to decrease peaks and pre- and post-prandial insulin concentrations in T2D subjects and also in people with impaired glucose tolerance [54].

Prior et al. have shown that an increased capillarization is associated with an improvement in glucose tolerance and insulin sensitivity measured during hyperinsulinemic euglycemic clamping. This study confirmed that 6 months of aerobic PA can improve capillarization in muscle fibres by 15%, leading to higher and more efficient exchanges, as well as a decrease in body weight [55].

Transcapillary insulin transfer is one of the mechanisms through which skeletal muscle achieves higher glucose absorption. A sedentary lifestyle intensify this risk through changes in insulin signalling pathways and changes in skeletal muscle morphology leading to a worsening of insulin resistance [56].

A meta-analysis assessed the effects of structured and programmed exercise in T2D subjects. Data from 14 clinical trials, lasting more than 8 weeks, comparing changes in HbA1 on cardiovascular risk factors (CVD risk) were evaluated. HbA1c levels were significantly lower in the group “treated” with PA compared to the control group. Data processing confirmed that the beneficial effects of PA on HbA1c values were independent from the effects on body weight. This study provides valid explanations for prescribing intensive aerobic exercise to patients with T2D as a treatment tool [57]. Najafipour et al. evaluated the effects of regular exercise, lasting about 8 years, on the health promotion in T2D patients. Participants performed 3 sessions of 90 min per week of regular PA. At the end of the trial, authors showed the direct relationship between long-term regular PA and HbA1c level and cardiovascular fitness, especially in terms of maximal oxygen uptake (VO_2_ max) and BMI [58].

Both in the general population and in individuals with diabetes, an inverse association between increased PA and lower risk of cardiovascular disease has been well assessed [59]. Nevertheless, guidelines for the recommended level of PA in T2D subjects suggest performing 30 min of moderate aerobic activity, such as walking at a sustained rate most days of the week in association with increased daily activities, or walking during breaks on working days, or performing domestic activities such as climbing stairs.

Many epidemiological studies demonstrate a close correlation between energy expenditure due to PA and reduced incidence of T2D. Data produced by the Nurses’ Health Study [60] have evaluated PA levels using a validated questionnaire. This study showed that the reduction of diabetes risk was associated with modest increase in PA [60]. Comparable results have also been observed by other epidemiological studies where moderate intensity of PA compared to inactivity reduces the incidence of new cases of DM by about 60% [59].

PA is an effective cost-saving tool in the treatment of T2D. Indeed, it has been established that 2 years of aerobic exercise, carried out as walking, have provided valuable benefits to the health of T2D subjects and considerable savings to the national health system [61].

**Figure 2:**
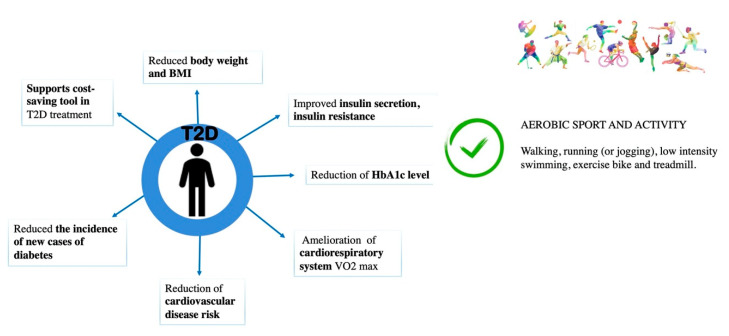
Beneficial effects of PA and recommended sports for T2D subjects.

Major beneficial effects of PA and recommended sports in T2D subjects are shown in Figure 2.

## 5. Discussion

The objective of non-pharmacological therapy of DM is to change lifestyle by promoting PA and diet. Beneficial effects produced by PA are different in T1D and T2D subjects due to differences in morphotype, aetiology and clinical and pharmacological treatment.

Indeed, PA may have different results in patients affected by T1D and T2D. T1D patients are younger, more active and more prone to PA, which can enhance their insulin sensitivity, glycaemic control, lipid profile, antioxidant defences and renal function and also decrease blood pressure and cardiovascular diseases. Otherwise, T2D patients are adults, who are usually affected by multiple comorbidities such as obesity, metabolic syndrome and musculoskeletal disorders and have a more sedentary life. The therapeutic effect of PA on these patients has been proved to be related to a better control of glucidic and lipidic metabolism, also on blood pressure and cardiovascular diseases, as well as reduction of BMI and an increase of insulin sensitivity in skeletal muscle.

The recommended activities are all pure or predominantly aerobic with the recommendation to avoid a competitive engagement longer than one hour [30]. It is advisable to use a cardiofrequency device that allows instantaneous measurement of the heart rate and to set audible alarms if the set frequency is exceeded. The exercise session should last 30 to 60 min, and blood glucose could be checked one hour after exercise to decide whether or not to take carbohydrates. Anaerobic sports or sports characterized by isometric or strength exercises, fighting sports with physical contact, activities involving frequent head shaking and scuba diving require special caution and should be carefully evaluated on a case-by-case basis.

Moreover, there is a strong link between mental and physical health. Several studies have found a clear relationship between PA, the quality of life and the psychological well-being. Indeed, PA is a major health behaviour with positive effects on mood, self-esteem, cognitive functioning, depression and quality of life, strongly recommended for the prevention and treatment of several non-communicable diseases [62,63].

PA is now firmly accepted as an effective non-pharmacological treatment of T2D [55], although the specific mechanisms underlying the positive effects of exercise remain unclear. Regular involvement of individuals with diabetes in exercise programs could become a potential way to improve their quality of life reducing the economic expenditure for diabetes treatment and reducing complications that result from it.

## 6. Conclusions

The goal of DM therapy is certainly to reduce the risk of short-and long-term complications. Drug therapy has beneficial effects on the risk of complications, but it is not sufficient to reverse them. The strongest indication shared by the most recent guidelines and consensus documents on the management of diabetic disease requires continuous attention to the implementation of a correct lifestyle and the necessity of therapy personalization, with the adaptation of pharmacological and non-pharmacological prescriptions (nutritional therapy, PA indications) to the metabolic and clinical profile of the individual patient.

## Figures and Tables

**Table 1:** Differences in physical activity (PA) types and their recommendation in type 1 (T1D) and type 2 diabetes (T2D) subjects.

Subjects	Aerobic	Resistance	HIIT
T1D	↑ lipid metabolism ↓ insulin resistance	↑ muscular strength ↑ lipid profile ↑ better control of blood glucose levels ↓ dose of insulin	↑ cardioprotective ↑ metabolic benefits
Recommendations	150 min/week at moderate to vigorous intensity	Engaging in 2–3 non-consecutive days/week	In younger when vigorously performed for 75 min/week.
T2D	↓ blood pressure ↓ triglycerides ↓ insulin resistance ↓ A1C	↑ blood pressure ↑ muscle mass and strength ↑ insulin responsiveness ↑ metabolic control ↑ lipid profile ↑ cardiovascular disease	↑ insulin sensitivity ↑ metabolic control
Recommendations	150 min/week at moderate to vigorous intensity	Perform both flexibility and balance activities for 2 or more sessions/week.	In physically fit patients when vigorously performed for 75 min/week.

**Legends** ↑ increase–↓ reduction.
